# Cerebrospinal fluid dynamics and brain function regulation: from homeostasis to neurological disorders

**DOI:** 10.3389/fnins.2026.1775240

**Published:** 2026-02-10

**Authors:** Yu Yang, Huixia Jia, He Liu

**Affiliations:** 1 Department of Systems Science, Faculty of Arts and Sciences, Beijing Normal University, Zhuhai, China; 2 International Academic Center of Complex Systems, Beijing Normal University, Zhuhai, China; 3 School of Systems Science, Beijing Normal University, Beijing, China

**Keywords:** brain function, cerebrospinal fluid, clinical medicine, fluid dynamics, neuroscience

## Abstract

Cerebrospinal fluid (CSF) is increasingly recognized as an active regulator of brain function rather than a passive mechanical buffer. Beyond its classical roles in cushioning the brain and removing metabolic waste, CSF participates in a tightly coupled system linking neural activity, vascular dynamics, molecular signaling, and tissue mechanics. Here, we present an integrated theoretical framework that unifies three major conceptual strategies in contemporary CSF research: metabolic clearance, neuromodulatory signaling, and bidirectional coupling between fluid dynamics and neural activity. We argue that these processes form a closed-loop regulatory system in which brain state governs CSF flow, while CSF dynamics reciprocally shape neural function and long-term brain health. Disruptions to this integrated CSF-brain system underlie a wide spectrum of neurological disorders, including Alzheimer’s disease, stroke, sleep disorders, and hydrocephalus. By synthesizing evidence across scales and disciplines, this framework provides a coherent conceptual foundation for future experimental, diagnostic, and therapeutic advances targeting CSF physiology.

## Introduction

For much of the twentieth century, cerebrospinal fluid (CSF) was regarded as a biologically inert medium whose primary functions were mechanical protection and waste removal (Nabiuni et al., 2012; Gato et al., 2005). This reductionist view is no longer tenable. Advances in neuroimaging, molecular neuroscience, and systems physiology have revealed CSF as a dynamic circulatory and signaling system that is deeply integrated with neural activity, vascular pulsatility, and astroglial function (Jessen et al., 2015; Iliff et al., 2012). Rather than serving merely as background fluid, CSF actively participates in maintaining brain homeostasis, coordinating metabolic processes, and modulating neural circuits.

A central insight driving this paradigm shift is the recognition that CSF dynamics are state-dependent. Cardiac pulsation and respiration provide fundamental drivers of fluid movement (Kiviniemi et al., 2016), but brain-state transitions, particularly the sleep-wake cycle profoundly shape the spatiotemporal patterns of CSF flow (Fultz et al., 2019). These patterns, in turn, regulate solute transport (Iliff et al., 2012), molecular signaling (Nedergaard and Goldman, 2020), and mechanical coupling between brain tissue and its surrounding fluid environment. Understanding CSF function therefore requires a systems-level perspective that integrates fluid dynamics, cellular mechanisms, and electrophysiology.

**FIGURE 1:**
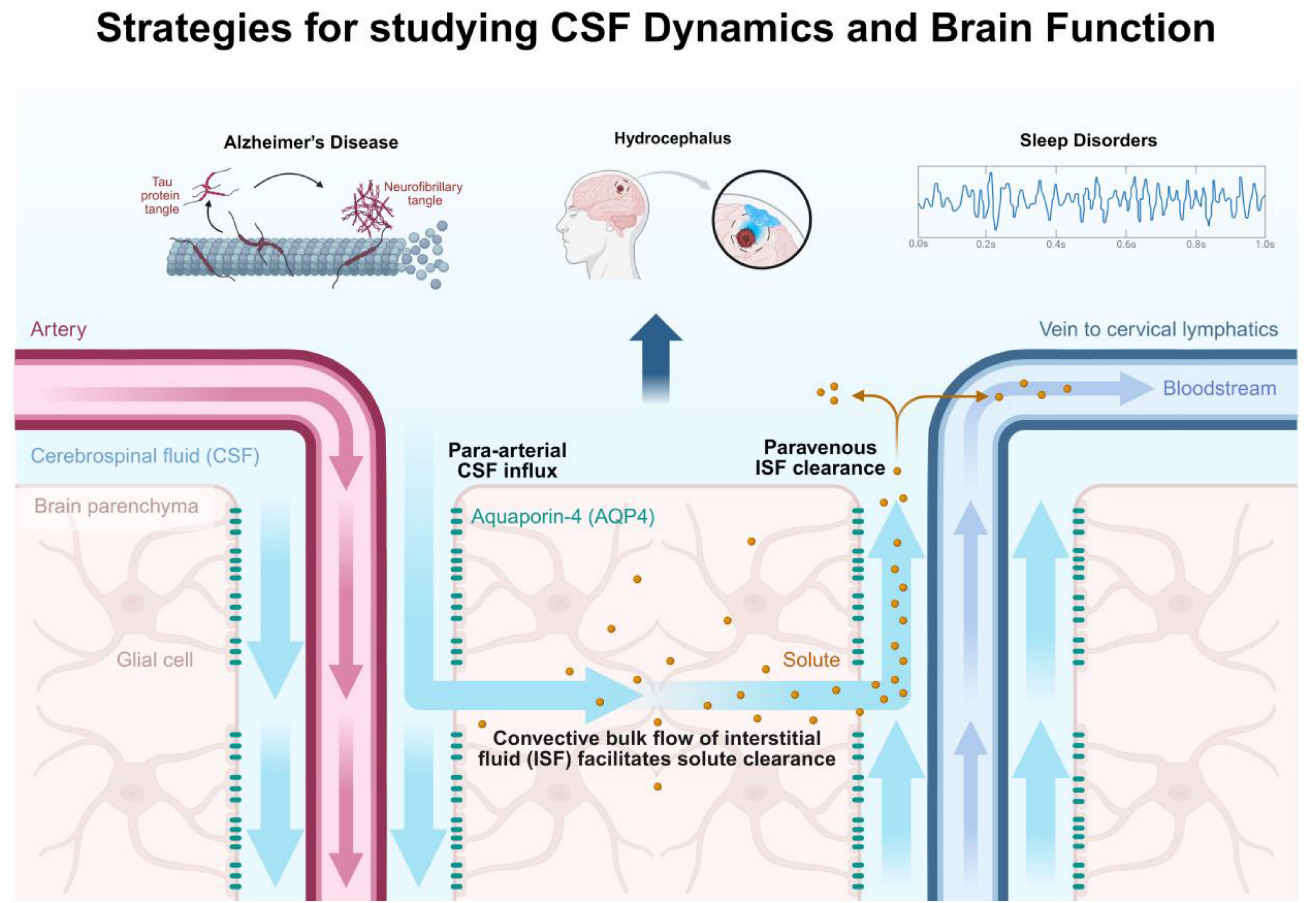
Strategies for studying CSF dynamics and brain function. Schematic overview of CSF production and circulation, the glymphatic clearance pathway, CSF-brain signaling interfaces, and clinical applications in neurological disorders.

In this manuscript, we propose an integrated conceptual framework that unifies three dominant strategies in CSF research (Figure 1): (i) metabolic clearance via glymphatic transport (Iliff et al., 2012; Hablitz et al., 2020), (ii) neuromodulatory signaling through CSF-borne molecules (Nedergaard and Goldman, 2020; Myung et al., 2018), and (iii) bidirectional coupling between CSF dynamics and neural activity (Fultz et al., 2019; Bojarskaite et al., 2020). We argue that these strategies represent interdependent components of a single regulatory loop rather than independent phenomena. This unified view clarifies the pathophysiological basis of diverse neurological disorders from Alzheimer’s disease, where impaired glymphatic clearance is linked to sleep disruption and amyloid-β accumulation (Ju et al., 2014; Holth et al., 2019), to idiopathic intracranial hypertension and normal pressure hydrocephalus, where altered fluid dynamics directly impact neural function (de Souza Bezerra et al., 2018) and highlights new opportunities for diagnosis and therapy.

## CSF production, circulation, and state-dependent dynamics

Cerebrospinal fluid is primarily produced by the choroid plexus at a rate of approximately 500–600 mL per day in adults, resulting in complete turnover several times daily (Oreskovic and Klarica, 2010; Brinker et al., 2014). From the ventricular system, CSF circulates through subarachnoid spaces and perivascular compartments before being absorbed via arachnoid granulations and meningeal lymphatic pathways (Jessen et al., 2015; Iliff et al., 2012). Importantly, this circulation is not passive. CSF motion is driven by arterial pulsatility, respiratory pressure gradients, and slow volumetric changes in brain tissue associated with neural activity (Plog and Nedergaard, 2018; Ringstad et al., 2017; Mestre et al., 2018; Dreha-Kulaczewski et al., 2015).

Sleep represents a critical modulatory state for CSF dynamics. During slow-wave sleep, reductions in noradrenergic tone lead to expansion of the interstitial space, facilitating increased CSF influx and enhanced solute transport (Kiviniemi et al., 2016; Lee et al., 2015). These state-dependent changes underscore the principle that CSF flow is actively regulated by brain physiology rather than imposed solely by peripheral forces (Kiviniemi et al., 2016; Ringstad et al., 2017).

## Metabolic clearance as an emergent systems process

The discovery of the glymphatic system provided a mechanistic explanation for how CSF participates in metabolic waste removal (Iliff et al., 2013a; Xie et al., 2013). Convective CSF flow along perivascular spaces enables the clearance of interstitial solutes, including amyloid-β and tau (Nedergaard and Goldman, 2020; Iliff et al., 2013a; Abbott et al., 2018). Astrocytic aquaporin-4 (AQP4) channels, polarized at perivascular end-feet, play a critical role in facilitating fluid exchange between CSF and interstitial compartments (Harrison et al., 2018; Zeppenfeld et al., 2017).

Within the integrated framework proposed here, glymphatic clearance is not an isolated function but an emergent property of coordinated neural, vascular, and glial activity (Nedergaard and Goldman, 2020; Rasmussen et al., 2022; Simon and Iliff, 2016). Neural oscillations and vascular pulsatility drive CSF motion, while astrocytic regulation of water permeability tunes exchange efficiency (Hablitz et al., 2020; Munk et al., 2019; Rasmussen et al., 2022). Disruptions at any level, sleep fragmentation, vascular stiffening, or loss of AQP4 polarity can impair clearance and promote pathological protein accumulation (Nedergaard and Goldman, 2020; Munk et al., 2019; Zeppenfeld et al., 2017; Simon and Iliff, 2016).

## CSF as a neuromodulatory and signaling medium

In addition to transporting solutes, CSF serves as a distributed signaling medium containing hormones, growth factors, cytokines, metabolites, and extracellular vesicles (Lehtinen et al., 2011; Wu et al., 2020; Candelario and Steindler, 2014). The molecular composition of CSF varies with circadian rhythm, behavioral state, and disease, reflecting ongoing neural and systemic processes (Blennow and Zetterberg, 2018; Johanson et al., 2008; Sankowski et al., 2015). Factors such as insulin-like growth factor-1 and brain-derived neurotrophic factor link CSF composition to synaptic plasticity, myelination, and cognitive function (Lehtinen et al., 2011; Louveau et al., 2015; Da Mesquita et al., 2018).

Crucially, CSF signaling feeds back onto neural circuits, influencing excitability and network synchronization (Johanson et al., 2008; Sankowski et al., 2015; Simon et al., 2017). Through this feedback, CSF composition can modulate the same brain states that govern its own circulation, embedding signaling within the broader CSF-brain regulatory loop (Blennow and Zetterberg, 2018; Louveau et al., 2015; Wu et al., 2020).

## Bidirectional coupling between CSF dynamics and neural activity

Emerging evidence demonstrates tight coupling between CSF motion and neural oscillations (Fultz et al., 2019; Hablitz and Nedergaard, 2021). Functional imaging studies reveal coordinated fluctuations in CSF flow, cerebral blood volume, and electrophysiological activity, particularly during sleep (Thomas, 2019; Iliff et al., 2013b; Kedarasetti et al., 2020). These observations suggest that CSF dynamics are actively synchronized with neural rhythms to optimize metabolic clearance and molecular transport (Fultz et al., 2019; Holter et al., 2017; van Veluw et al., 2020).

Pathological alterations in CSF dynamics directly perturb neural function (Mortensen et al., 2019). In normal pressure hydrocephalus, abnormal CSF pulsatility is associated with slowed cortical rhythms and cognitive impairment, which can be partially reversed by restoring CSF flow (Holter et al., 2017; Asgari et al., 2016; Mortensen et al., 2019). Such findings highlight bidirectional coupling as the organizing principle linking clearance and signaling within a unified system (Fultz et al., 2019; van Veluw et al., 2020; Hablitz and Nedergaard, 2021; Winer et al., 2019).

## Neurological disorders as failures of the integrated CSF-brain system

From this systems perspective, neurological diseases can be reinterpreted as breakdowns of integrated CSF-brain regulation (Tarasoff-Conway et al., 2015; de Leon et al., 2017; Bothwell et al., 2019). In Alzheimer’s disease, impaired state-dependent CSF flow and glymphatic clearance promote toxic protein accumulation (Nedergaard and Goldman, 2020; Tarasoff-Conway et al., 2015; Reeves et al., 2020), while altered CSF signaling further disrupts synaptic function (de Leon et al., 2017; Yun et al., 2020). In stroke and traumatic brain injury, dysregulated CSF dynamics contribute to cerebral edema and secondary injury (Yun et al., 2020; Gaberel et al., 2014). Sleep disorders impair CSF-mediated clearance, potentially accelerating neurodegeneration (Lee et al., 2015; Xie et al., 2013) while hydrocephalus represents a global failure of CSF circulation and absorption (Strahle et al., 2011; Karimy et al., 2017; Jiang et al., 2017).

Viewing these conditions through a unified framework emphasizes shared mechanisms and suggests that therapeutic interventions targeting CSF dynamics may yield broad benefits across traditionally distinct disorders (Tarasoff-Conway et al., 2015; Reeves et al., 2020; Bothwell et al., 2019).

## Technological and therapeutic implications

The emergence of an integrated CSF-brain framework has been accompanied by rapid technological advances that now make it possible to interrogate, model, and manipulate CSF dynamics with unprecedented precision. These developments are accelerating the translation of conceptual insights into clinical and therapeutic applications.

Advanced Imaging and Quantification of CSF Dynamics: non-invasive neuroimaging has become central to characterizing CSF circulation and its coupling to brain activity. Phase-contrast MRI enables quantitative measurement of CSF flow velocities and pulsatility across ventricular and subarachnoid compartments (Battal et al., 2011), while time-resolved three-dimensional sequences provide spatial maps of flow vectors (Yamada et al., 2008). Diffusion-based techniques, including tensor-valued diffusion encoding, allow indirect assessment of perivascular space geometry and glymphatic transport efficiency (Schirge et al., 2025; Taoka et al., 2017). When combined with functional MRI and electroencephalography, these approaches enable simultaneous mapping of neural activity, vascular dynamics, and CSF motion, offering a systems-level view of fluid-brain interactions (Fultz et al., 2019).

Emerging ultra-fast imaging sequences and low-dose contrast protocols hold promise for capturing state-dependent CSF dynamics in humans, including sleep-associated oscillations that were previously accessible only in animal models (Ringstad et al., 2017; Lee et al., 2015). Such advances are essential for validating glymphatic function as a clinically relevant biomarker (Iliff et al., 2012).

Implantable and Wearable Monitoring Technologies: Miniaturized, wireless implantable sensors are transforming the monitoring of intracranial pressure, CSF composition, and biochemical markers in real time (Deng et al., 2025; Zhou et al., 2025). These devices allow continuous assessment of CSF dynamics in patients with hydrocephalus, traumatic brain injury, or subarachnoid hemorrhage, enabling personalized and adaptive management strategies. Parallel advances in wearable sleep and respiration monitoring provide complementary data on physiological drivers of CSF flow, facilitating integrated analysis across behavioral and fluid-dynamic domains (Chong et al., 2022).

Computational Modeling and Digital Twins: computational models integrating fluid mechanics, tissue biomechanics, vascular dynamics, and electrophysiology are increasingly used to interpret experimental data and predict therapeutic outcomes (Lakin et al., 2003; Vinje et al., 2019). Patient-specific models derived from imaging data enable simulation of CSF flow under different physiological and pathological conditions, supporting surgical planning and optimization of shunt placement in hydrocephalus (Sweetman and Linninger, 2011; Spijkerman et al., 2019). More broadly, the development of “digital twin” models of the CSF-brain system may allow *in silico* testing of interventions aimed at restoring normal fluid-neural coupling (Kissas et al., 2020).

Cerebrospinal fluid-Targeted Therapeutic Strategies: the recognition of CSF as an active regulatory medium has opened new therapeutic avenues. Pharmacological modulation of CSF production at the choroid plexus, for example through targeting ion transporters or metabolic pathways (Damkier et al., 2013), offers alternatives to purely mechanical interventions. Modulation of astrocytic AQP4 expression or polarization represents another promising strategy to enhance glymphatic clearance (Iliff et al., 2012) or control cerebral edema (Papadopoulos et al., 2004), though achieving spatial and temporal specificity remains a challenge (Rasmussen et al., 2022).

Beyond modulation, the CSF circulation itself is being harnessed as a therapeutic delivery route. Intrathecal and intraventricular drug delivery bypass the blood brain barrier and enable global distribution of small molecules, biologics, and gene therapy vectors (Pardridge, 2020; Bleyer and Poplack, 1979; Peyrl et al., 2014; Maurizi et al., 2014; Al Shaer et al., 2024; Greenberg et al., 2022; D’Avanzo et al., 2020). Convection-enhanced delivery (Vogelbaum and Aghi, 2015) and nanoparticle-based carriers (Ekhator et al., 2023) further exploit CSF flow patterns to improve targeting efficiency and reduce systemic toxicity. These approaches are particularly attractive for diffuse neurodegenerative diseases (Kariolis et al., 2020) and leptomeningeal pathologies (Glantz et al., 1999).

Neuromodulation and State-Based Interventions: non-invasive neuromodulatory techniques, including transcranial electrical and magnetic stimulation, are increasingly explored as tools to influence CSF dynamics indirectly by altering neural and vascular rhythms (Rasmussen et al., 2022). By entraining slow oscillations or modifying sleep architecture, such interventions may enhance glymphatic clearance and optimize CSF-mediated signaling (Xie et al., 2013). Behavioral interventions, especially sleep optimization and respiratory therapy, represent low-risk strategies that directly leverage physiological drivers of CSF flow (Holth et al., 2017; Lilius et al., 2019).

Collectively, these technological and therapeutic developments reflect a shift from treating CSF abnormalities as isolated mechanical problems to targeting the CSF-brain system as an integrated, dynamic regulator of neural health.

## Conclusion

Cerebrospinal fluid is a central component of an integrated brain regulatory system that links metabolism, signaling, and mechanics through bidirectional coupling with neural activity. Recognizing clearance, neuromodulation, and fluid-neural interactions as elements of a single closed-loop framework provides a coherent theoretical foundation for future research. By targeting CSF physiology as a systems-level process, new strategies may emerge for preserving brain health and treating neurological disease.

## Data Availability

The original contributions presented in this study are included in this article/supplementary material, further inquiries can be directed to the corresponding author.
